# A Review of Recent Developments in Turner Syndrome Research

**DOI:** 10.3390/jcdd8110138

**Published:** 2021-10-23

**Authors:** Allen C. Huang, Susan B. Olson, Cheryl L. Maslen

**Affiliations:** 1 Medical School, Oregon Health & Science University, Portland, OR 97239, USA; huangall@ohsu.edu; 2 Department of Molecular and Medical Genetics, Oregon Health & Science University, Portland, OR 97239, USA; olsonsu@ohsu.edu; 3 Knight Cardiovascular Institute, Oregon Health & Science University, Portland, OR 97239, USA

**Keywords:** Turner syndrome, cardiovascular, genetics, epigenetics

## Abstract

Turner syndrome is a rare disorder resulting from complete or partial loss of the second sex chromosome. Common manifestations include delayed growth, premature ovarian failure, congenital heart defects, endocrine disorders, lymphedema, and webbed neck. People with Turner syndrome have significantly increased mortality risk primarily due to cardiovascular abnormalities. The mechanisms that lead to these defects are not completely understood and are obscured by the significant variability of both karyotype and phenotype without consistent correlation between the two. This paper presents a review of the recent literature surrounding the symptoms, mechanisms, diagnosis, and treatment of Turner syndrome with a focus on cardiovascular manifestations. With technological advancements in genetics, the molecular processes of Turner syndrome have begun to be dissected. Certain genes on the X chromosome that typically escape inactivation have been implicated in both specific manifestations and broader risk categories. Recently identified genome-wide epigenetic changes may help explain the variability in presentation. It remains unclear as to how the combination of these factors results in the overall clinical picture, but advances in genomic, genetic, epigenetic, and -omics technology hold promise for providing insights that will improve the medical management of individuals with Turner syndrome.

## 1. Introduction

Turner syndrome is a spectrum of phenotypic characteristics that result from deficiency of second sex chromosome genes. It is estimated to occur once per roughly 2500 female live births [1]. The most common manifestations are short stature, which is usually recognizable by age 5, and early loss of ovarian function, which usually prevents puberty and causes infertility. Additional symptoms vary and include skeletal abnormalities, lymphedema, web neck, and congenital heart defects [2]. Cardiovascular abnormalities are of significant concern both in utero and postnatally. Heart defects incompatible with life contribute to an estimated 99% fetal loss [3], while bicuspid aortic valve and coarctation of the aorta are more common complications of live births [4]. Later in life, aortic aneurysm and dissection at relatively young ages contribute to increased mortality. In young adults, there is a significantly higher incidence of hypertension and type 2 diabetes mellitus than seen in the general population [5]. These diseases alone contribute to a threefold increase in early mortality compared to the euploid population [4]. Current standard management includes growth hormone and estrogen therapy to maintain normal growth and development. Additionally, congenital heart defects are screened for and corrected early in life. However, the median age of diagnosis is around 15 years [1], presenting an issue for early treatment.

**Figure 1:**
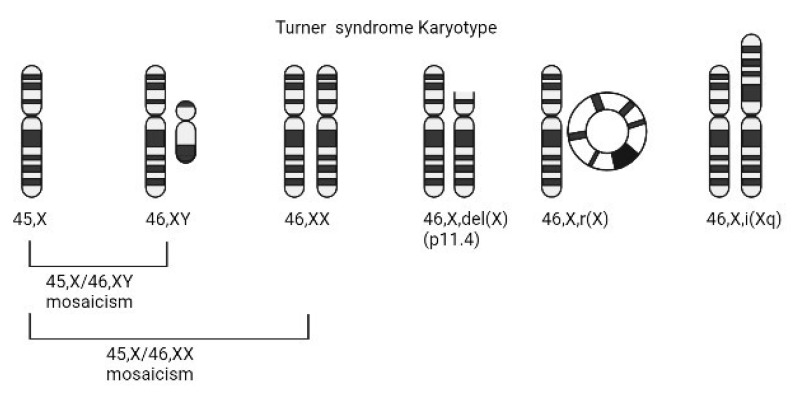
Common Turner syndrome sex chromosome karyotypes with complete or partial loss of genetic material on the second sex chromosome. The ideograms, left to right, represent: monosomy X, mosaicism, X short arm deletion, ring chromosome X, and isochromosome X as the most common karyotypes in Turner syndrome. 45,X/46,XY mosaicism may involve a structurally abnormal Y chromosome.

Despite being identified over a century ago, the exact etiology of most Turner syndrome manifestations remains unclear. This is due in part to the fact that not all individuals with Turner syndrome have the same sex chromosome composition. Around 50% of cases are the result of a completely missing second sex chromosome (45,X) [5]. Other common karyotypes include 45,X/46,XX mosaicism, isochromosomes of the Xp or Xq, ring X chromosomes, and the presence a Y chromosome (Figure 1). As a result, individuals with Turner syndrome are phenotypically female even though they might have been destined to be male until early loss of the Y chromosome. However, in spite of the fact that 50% of individuals with Turner syndrome have the same 45,X sex chromosome constitution, phenotypic variability is high and cannot be fully accounted for by sex chromosome variation. This has created both challenges and opportunities in identifying the dysregulated genes that impact growth and development and result in the variable and complex Turner syndrome phenotype.

## 2. Diagnosis

A diagnosis of Turner syndrome is made by cytogenetic testing. Historically, diagnosis has been in a multimodal age distribution with peaks at birth, during puberty, and in later adulthood, with a median age of diagnosis around 15 years [1]. Turner syndrome may be suspected through routine ultrasound surveys or other prenatal screening methods. The availability of cell-free DNA (cfDNA) screening has aided in early detection [6], however, it has a limited positive predictive value due to mosaicism and contamination from maternal DNA. Prenatal karyotype analysis through chorionic villus sampling or amniocentesis is necessary to confirm the diagnosis. The gold standard has been G-banded chromosome analysis with the use of FISH with X- and Y-specific probes when mosaicism is suspected. Recently, chromosomal microarray using oligonucleotide and SNP platforms has been shown to be equivalent in making the diagnosis in patients with Turner syndrome, with the exception of low-level mosaicism [7].

## 3. Management

The primary treatment for patients with Turner syndrome is growth hormone therapy to maintain body stature [8,9]. While GH has been shown to be effective at increasing the height of patients with Turner syndrome, variables such as dosing and late initiation of treatment can reduce efficacy [2]. Additionally, estrogen replacement therapy is important due to the increased risk for premature ovarian failure. Estrogen is critical for the induction of puberty, and many patients with Turner syndrome who are not treated have delayed puberty and amenorrhea. Even after puberty, patients with Turner syndrome are at high risk for early menopause due to premature ovarian failure and benefit from extended hormone replacement. While some patients are spontaneously fertile, the majority require oocyte donation if they wish to pursue pregnancy [5], which is possible but is generally high-risk due to cardiovascular risk factors. Exogenous estrogen has protective effects on bone density, cardiovascular health, blood pressure, neurocognition, and sexual function [2]. Even when receiving GH and/or hormone replacement therapy, patients with Turner syndrome need multidisciplinary follow-up due to their increased risk for cardiovascular, metabolic, orthopedic, renal, and neuropsychological abnormalities.

All patients with Turner syndrome should have a screening trans-thoracic echocardiogram at the time of diagnosis with a follow up cardiovascular MRI if the echocardiogram does not produce adequate images. Cardiovascular CT may be used if cardiovascular MRI is contraindicated. It is highly recommended that patients with Turner syndrome have periodic cardiovascular imaging due to increased risk of aortopathy. As patients with Turner syndrome age, the acoustic window of the chest cavity changes due to a barrel shaped chest and echocardiogram becomes more difficult [10]. Because of the abnormal chest shape and increased risk for aortopathy, it is recommended that cardiovascular CT or MRI is performed every 5 to 10 years even in asymptomatic patients to monitor the aorta for dissection.

## 4. Cardiovascular Manifestations

Congenital heart defects, which occur in about 25–50% of cases, are the most common cause of death in patients with Turner syndrome [4]. However, diagnosis can be challenging as imaging of the heart and aorta are more difficult due to differences of the chest wall morphology [10]. Occurring at a rate of up to 30%, bicuspid aortic valve (BAV) is the most common congenital malformation in Turner syndrome [11]. BAV occurs when two of the leaflets of the aortic valve fail to separate during embryogenesis, resulting in increased risk of both valvular and aortic pathologies such as aortic insufficiency, aortic aneurysm, and aortic dissection [12]. In the general population, BAV is the most common congenital heart defect with an incidence of 0.5–2%, with males being affected three times more often than females [12]. Hence, BAV is approximately 60 times more likely to be seen in an individual with Turner syndrome than a euploid female. In fact, it has been hypothesized that the presence of BAV in female infants may help in an earlier diagnosis of Turner syndrome as an indicator that a karyotype should be performed [4].

BAV is also associated with aortopathies such as coarctation of the aorta (CoA), which can occur in up to 18% of patients [3]. CoA and BAV can also occur independently. CoA is an abnormal narrowing of the aortic arch and is also associated with aneurysm and dissection as well as hypertension. It has been established that the 45,X karyotype has a higher incidence of congenital abnormalities than other karyotypes. This suggests that there may be specific genes on the X chromosome that are protective against congenital heart defects when present [3,4], which is consistent with the increased rate of BAV in euploid males. BAV and coarctation of the aorta are most commonly seen in live-born cases of Turner syndrome because they are survivable, whereas other more severe congenital heart defects contribute to the high rate of fetal loss. Less common defects in surviving individuals include venous abnormalities, atrial and ventricular septal defects, hypoplastic left heart syndrome, and mitral and pulmonary valve abnormalities [3]. An association with arrythmias including prolonged QT interval has also been noted [13]. Coronary artery abnormalities including absent left main coronary artery are often asymptomatic but affect surgical courses. Fetal echocardiograms should be performed if Turner syndrome is suspected prenatally, and transthoracic echocardiogram should be performed for all patients with Turner syndrome to rule out congenital heart disease [4].

**Figure 2:**
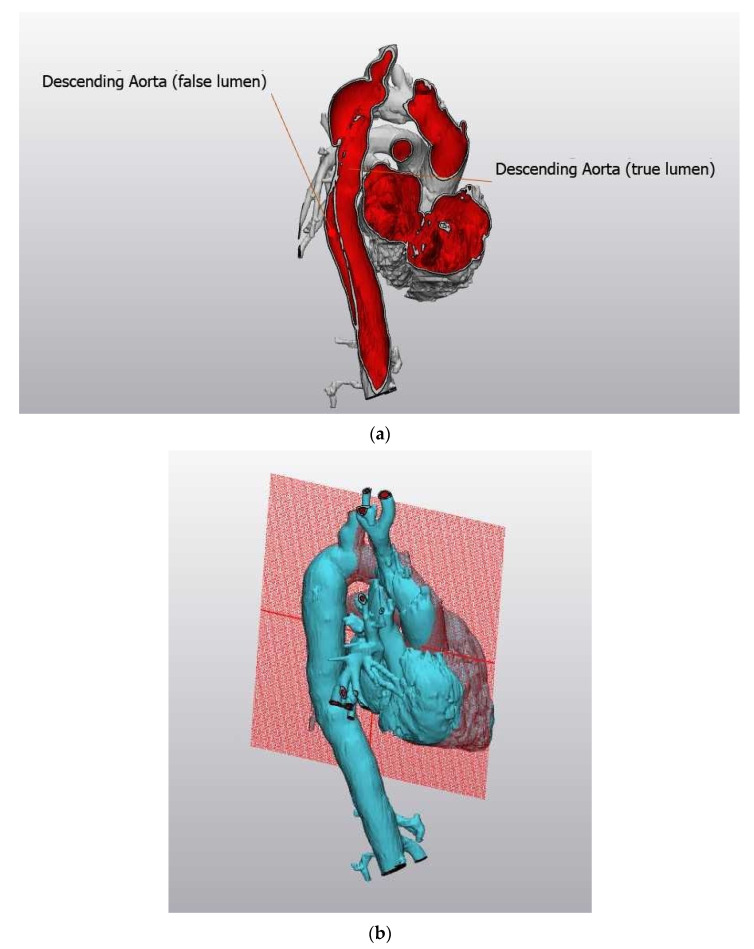
(**a**) Three-dimensional reconstruction of aortic dissection that occurred in a young woman with Turner syndrome. (**b**) Plane of reference for Figure 2a.

Due to the increased rate of congenital defects, there is a higher incidence of associated cardiovascular complications including aortic aneurysm and dissection (Figure 2a,b, courtesy of Dr. Michael Silberbach). Figure 2a shows an image of an aortic aneurysm of the descending aorta creating a false lumen, threatening the life of a young woman with Turner syndrome. Figure 2b shows the plane of reference for Figure 2a.

Dissection occurs at a 100-times greater rate and at younger ages in the Turner syndrome population than the general population [3]. In the Turner syndrome population, the median age of dissection was found to be 30–35, as opposed to 70 in the general population [14,15]. The increased risk of aortic pathology is partially attributed to congenital defects, but there are also additional factors specific to Turner syndrome that lead to elevated risk as there is evidence that aortopathy occurs in Turner syndrome in the absence of BAV [4,16]. Decreased estrogen levels and increased arterial wall stiffness and thickness have been suggested as significant contributing factors [3].

In addition to structural abnormalities, there is also increased risk for adult-onset cardiovascular metabolic disease in Turner syndrome. Blood pressure is elevated in both pediatric and adult populations, with up to 40% of children and 60% of adults being affected [3,17]. The cause of hypertension is not known, but it is suspected to be multifactorial stemming from decreased estrogen, aortopathy, and sympathetic dysregulation [2]. Estrogen replacement therapy has been shown to be beneficial in lowering blood pressure [2]. Patients with Turner syndrome are also at increased risk for type 1 and type 2 diabetes through an unknown mechanism. There are more specific implications of a genetic link in diabetes, as the rate of diabetes depends on the genotype. Patients with isochromosome Xq had highest rates, followed by Xp deletions and complete deletions. Patients with Xq deletions had rates similar to the general population [18]. Growth hormone therapy further decreases insulin sensitivity, compounding risk for diabetes mellitus [19]. The 45,X karyotype is also associated with hypercholesterolemia [20]. Patients with Turner syndrome are also at increased risk for ischemic heart disease and stroke. As with hypertension, the cause of these events is not established, but it is again believed to stem from the combination of factors including estrogen deficiency, dyslipidemia, hypertension, and diabetes. With so many risk factors, the mortality of Turner syndrome patients is three times as high as euploid individuals [4]. This increased mortality is well explained by the increased rates of previously mentioned pathologies (Table 1).

## 5. Genetics

There are multiple karyotypes that cause Turner syndrome, each involving a significant deletion of the second sex chromosome. The range of karyotypes spans from complete loss of the X chromosome (45,X) to mosaicism (45,X/46,XX), as well as variants with isochromosome of the Xp or Xq arm, ring X chromosomes, and the presence of a cell line containing a Y chromosome (45,X/46,XY), in some cases with the Y being structurally abnormal. The importance of identifying a Y-containing cell line is that these individuals have an increased risk of developing gonadoblastoma [22]. An estimated 99% of 45,X karyotype fetuses are spontaneously aborted during the first trimester [23], and therefore it is possible that there is unidentified mosaicism in surviving patients that decreases phenotypic severity. The fact that each of the variations can be grouped into a syndrome with an overlapping constellation of phenotypic manifestations implicates the loss of sex chromosome genes in the pathogenesis of the disorder. Certain karyotypes have been associated with increased risk for specific comorbidities. The 45,X karyotype has increased risk of all-cause mortality as well as congenital heart abnormalities [3]. The 45,X, 45,X/46,X,i(Xq), and 46,X,r(X)/46,XX karyotypes have increased risk of renal defects [24]. Additionally, ring X karyotypes have been associated with increased risk of metabolic abnormalities [24]. Mosaic karyotypes may have decreased risk of cardiovascular abnormalities [25]. Even within 45,X karyotypes, the parental origin of the lone X chromosome may be significant, as patients with a maternal X chromosome had increased aortic stiffness compared to those with a paternal X chromosome [26]. However, the fact that there is significant variability even among patients with identical karyotypes confounds the understanding of the genetics behind Turner syndrome, and thus it appears that additional genetic variation likely plays a role in the final outcome, prompting research to identify genes and alterations in gene regulation associated with comorbid conditions in Turner syndrome [23]. In fact, researchers in the field now think of Turner syndrome not as a disease itself, but as a condition that predisposes affected individuals to a broad range of diseases with loss of sex chromosome genes acting as a sensitized genetic background [27,28].

Recent research has associated absence of specific genes with different aspects of Turner syndrome. In euploid 46,XX individuals, one X chromosome is silenced as proposed by the Lyon hypothesis. However, up to 15% of genes escape inactivation, and the absence of these escape genes may be implicated in the pathogenesis of Turner syndrome [29]. Xp gene dosage has been implicated in disease, further helping researchers localize specific genes [24]. This association was made after associating deletions of the short arm of the X chromosome, such as isochromosome Xq, as having increased comorbidities than deletions in the opposite arm. The first major breakthrough was the association of the *SHOX* Xp22.33 gene with short stature in Turner syndrome [30]. *SHOX* is also associated with Léri–Weill syndrome and Langer-type mesomelic dwarfism, which both share the characteristic finding of the lower limb being proportionally shorter than the upper limb. Using the homeobox *SHOX2* gene on chromosome band 3q25.32 and the orthologue *Og12x* in mice, researchers have determined SHOX to play a homeodomain role in development of limbs as well as the first and second pharyngeal arches. More recently, the *KDM6A* Xp11.3 gene (also known as *UTX*) has been associated with hyperinsulinism and hearing loss [31,32]. *KDM6A* is the gene mutated in Kabuki syndrome, which also presents with growth deficiency, cardiac abnormalities including left-sided lesions, aortopathy, metabolic abnormalities, and sensorineural hearing loss [32]. KDM6A has been shown to play a critical role in histone demethylation of genes critical for heart development, and lack of the gene has been associated with congenital heart defects and fetal lethality [33].

In another study using whole exome sequencing and a SKAT-O gene-based burden test, single nucleotide variants (SNVs) of *TIMP3* on chromosome band 22q12.3 were identified as having significant association with BAV and increased aortic dimensions as an indicator of aortic disease (BAVD) [34]. One dinucleotide polymorphism in particular accounted for the significant association. These two linked SNVs have been shown to reduce TIMP3 expression by about 60%. It is a common polymorphism in the general population and has not been associated with any disease phenotype in euploid individuals. *TIMP3* encodes a tissue inhibitor of matrix metalloproteinases (TIMPs), which is important in both regulation of valve formation and protection from aortic aneurysm. Notably, matrix metalloproteinases have been implicated in the growth of tumors, and TIMPs have been developed as pharmaceutical treatment [35]. The *TIMP1* Xp11.3 gene is a functionally redundant paralogue of *TIMP3*. In addition, *TIMP1* is known to escape X-inactivation and is polymorphically expressed [36]. Analysis of individuals with Turner syndrome with varying second sex chromosome karyotypes showed that the combined loss of *TIMP1* and the presence of the *TIMP3* risk allele significantly increased risk for BAVD. Increased copy number for *TIMP1* in mosaic individuals appeared to be protective. The overlapping functions of TIMP1 and TIMP3 in the developing heart valves and the aorta may explain why euploid individuals carrying the functional *TIMP3* dinucleotide variant are not at increased risk for BAV and aortopathy. Loss of TIMP3 expression may be compensated for by an increase in TIMP1, which cannot occur in Turner syndrome when there is hemizygosity of *TIMP1*. This suggests that some manifestations of Turner syndrome may be due to a two-hit mechanism, with loss of genetic material on the X chromosome combining with alterations elsewhere in the genome to cause pathology. It may also help explain why euploid males are at increased risk for BAVD compared to euploid females since males are also hemizygous for *TIMP1*.

GWAS data comparing individuals with Turner syndrome and left-sided heart defects to Turner syndrome with healthy hearts provides additional evidence that BAVD and other congenital heart defects are genetically heterogeneous. This study showed that a copy number variant (CNV) at 12p13.31 was associated with a significantly increased risk for left-sided heart lesions (including BAV) and aortic dissection [37]. In addition, this CNV was significantly over-represented in those with a 45,X chromosomal complement than other Turner syndrome karyotypes. It encompasses three protein coding genes, *SLC2A3*, *SLC2A14*, and *NANOGP1*. It is polymorphic and is seen in euploid individuals where it has been implicated as a contributing factor to the etiology of cardiac abnormalities in 22q11 deletion syndrome when duplicated in affected individuals. This CNV is also found in the general population but is not associated with disease except when it co-occurs with other gene deletions such as 45,X or 22q11 deletion. While the copy number polymorphism alone is not enough to increase risk, when present in a syndrome it may combine with other risk factors. The autosomal location of these genes further supports a two-hit hypothesis for congenital heart defects in Turner syndrome.

## 6. Epigenetics

**Figure 3:**
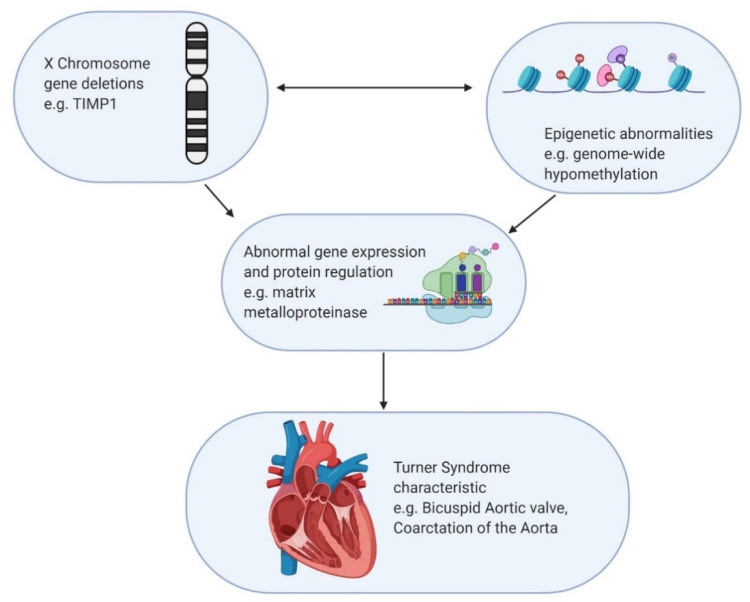
Hypothetical mechanism for interaction between genetic and epigenetic factors resulting in Turner manifestation. The example used involves loss of the *TIMP1* gene interacting with genome wide hypomethylation with unclear causation, resulting in cardiovascular defect.

Epigenetics encompasses the heritable patterns in gene expression that are not reflected in the DNA sequence. With advances in molecular biology, clinical research investigating the epigenetic mechanisms of disease presents as a largely untouched field of potential research. Common mechanisms of gene modification include CpG DNA methylation, histone acetylation, and noncoding RNA modification. A primary example of epigenetic mechanisms is the normal inactivation of one X chromosome in 46,XX individuals. This inactivation is accomplished with the expression of the *XIST* gene product, which is expressed exclusively by the inactivated X chromosome. As previously mentioned, the genes that escape inactivation potentially form the genetic basis for Turner syndrome when deleted. However, gene expression is also regulated by epigenetic mechanisms not isolated to the X chromosome (Figure 3), which may have implications for the variable phenotypic manifestations in Turner syndrome. It has been established that patients with Turner syndrome have different methylation status of both sex and autosomal chromosomes as well as distinct RNA-expression profiles. Specifically, Turner cells show genome-wide hypomethylation [38]. This global status allows for the possibility of genetic mechanisms of Turner syndrome to arise from other locations in the genome outside of the X chromosome.

Beyond establishing that there is a distinct genome expression profile in Turner cells, recent research has investigated how specific genes on the X chromosome may affect gene expression in other locations. The pseudoautosomal *CSF2RA* gene Xp22.32, previously linked to elevated intrauterine mortality, was found to be differentially methylated [2]. The *KDM6A* (*UTX*) gene at Xp11.3 encodes a histone H3K27 demethylase and has been linked to immune deficiency in Th cells [39]. The histone demethylase is also critical for promoting genes involved in development of heart cells, to the extent that embryonic stem cells without KDM6A failed to undergo proper differentiation. The *OGT* gene at Xq13.1 is involved in post-translational modification of proteins for a variety of cellular processes [40]. It is possible that genes such as *XIST* and *OGT* are involved in regulation of genes on different chromosomes, and the deletion of these genes in Turner syndrome may be critical in the Turner phenotype [41].

While it is possible that loss of material on the X chromosome causes abnormal regulation of genome-wide transcription, it is also possible that the causation is reversed [42]. It is theorized that a difference in methylation status of the sex chromosome could cause errors forming the spindle during meiosis or mitosis, leading to alignment errors [40]. It has been shown that cells with differential methylation status due to mutations in the DNA methyltransferase 3b gene had delayed centromere separation leading to aneuploidy [43]. Telomere length is another epigenetic marker may have an effect on meiotic disjunction and ovarian failure in Turner syndrome. Telomeres are an important aspect for alignment and synapse of homologous chromosomes during meiosis, and disruption can lead to errors potentially including one X chromosome self-synapsing [44]. Additionally, telomere length is implicated in reproductive aging, and could play a major role in the early ovarian failure seen in Turner syndrome. If the epigenetic profile seen in Turner syndrome is established to be the cause of nondisjunction, it would be a potential target for parental screening.

Significant research has been done in determining the parental origin of the remaining normal X chromosome in patients with Turner syndrome. As previously mentioned, increased aortic stiffness has been associated with a retained maternal X chromosome [26]. However, parental origin had no significant effect on rates of BAV, coarctation of the aorta, or response to replacement GH therapy [45]. It has been confirmed that the majority of patients with Turner syndrome have a maternal X chromosome at a rate of 60–80% [46]. Fetuses with a paternal chromosome may be subject to higher rates of fetal loss [47,48], but there is also evidence that the ratio of maternal to paternal chromosomes is skewed in utero [49]. Because there are more patients with a retained maternal chromosome, abnormal meiosis of the paternal chromosome may be a more significant driver of in the cause Turner syndrome. This could have implications in establishing risk factors such as advanced paternal age or sperm viability, neither of which have been linked to Turner syndrome. However, there is also evidence of Turner syndrome occurring from in vitro fertilization, which suggests that paternal meiosis is not the only potential error. Maternal meiotic errors, as well as mitotic errors in the early embryo, are other potential stages in which the nondisjunction could occur. Turner syndrome patients with a mosaic karyotype also demonstrate that not all cases are due to a meiotic error before fertilization. Errors in early zygote formation may allow for these findings.

## 7. Mouse Models

A major challenge in the investigation of genetics in Turner syndrome is the incorporation of a mouse model. In contrast to humans, mice with 39,X karyotype are phenotypically normal with no loss of fetal viability and normal fertility. In addition, although this mouse model has the equivalent of the Tuner syndrome karyotype, they do not exhibit stunted growth and do not show the same congenital cardiovascular abnormalities as humans with Turner syndrome. This difference between species is likely due to the fact that in contrast to humans, mice have relatively few genes on the X chromosome that escape inactivation, lessening the significance of an X chromosome loss [40]. Strains of mice have been bred specifically for study of Turner syndrome [50], and though the model is incomplete it has potential for replicating certain aspects of Turner syndrome pathology [51]. For example, mice have been effectively used to model certain aspects of Turner syndrome using the knockout of genes thought to be involved in specific Turner syndrome symtoms [30].

Recent research has attempted to determine the extent to which mice serve as a useful model for Turner syndrome. Although the number of escape genes are fewer, there is some overlap with genes of known significance in humans such as *KDM6A* [52]. In a genome-wide expression profile of brain, liver, kidney, and muscle, mice were also found to demonstrate tissue-specific expression and post-translational modification of X chromosome genes, an aspect that has not yet been explored in humans. The liver demonstrated the greatest differences in expression between 39,X and 40,XX mice, and some genes that were expressed in different amounts in the one tissue were not significantly different in another tissue [52]. It is not known if this profile is relevant to the clinical manifestations of Turner syndrome in humans as the differential expression has not been identified in human organs of interest such as the heart, vasculature, or ovaries. It has also been established that X gene dosage affects risk for angiotensin II-induced aortopathy in mice, regardless of ovariectomy [53]. X gene dosage has also been shown to affect body weight, metabolism, and adiposity [51]. In mice, the *Ogt* gene has been established to control histone methylation and has been linked to protection for fetal stress [50]. It has also been demonstrated that as in humans, mice with 39,X karyotype have epigenetic changes including genome-wide hypomethylation [40]. Like humans, mice demonstrate some difference with regards to parental origin of the remaining X chromosome. Mice with maternal X chromosomes had higher rates of angiotensin II-induced aortopathy and BAV than those with paternal X chromosomes or normal genotype [54]. The *Xlr3b* gene is imprinted and expressed at a greater level on the maternal X chromosome [54]. Although incomplete, the mouse model clearly has some potential in replicating both the genetic and epigenetic mechanisms of Turner syndrome.

## 8. Discussion

Turner syndrome is a complex disorder in which genetic and epigenetic factors lead to an associated group of clinical findings. The combination of factors leading to significant cardiovascular defects has received considerable attention in recent research due to significant mortality from defects such as aortopathies. With the identification of cardiovascular associated escape genes such as *TIMP1* and *KDM6A* on the X chromosome in recent research, more aspects of the underlying mechanisms of disease have begun to present themselves. Further investigating the relationship between these identified genes and the way their absence affects growth and development will be critical for future research. Understanding how specific gene dosage affects cellular function may play a key role in improving management and decreasing mortality. Future research may find it possible that the process leading to cardiovascular dysfunction is modifiable, though this remains unproven.

Beyond associations between karyotype and phenotype, analysis of epigenetic associations at both the chromosomal and genome level have gained recent traction in the literature. There is still very little research however on the interplay between genetics and epigenetics. In order for the most significant comorbidities such as BAV to be fully understood in Turner syndrome, more research will need to determine the interplay between genes such as *TIMP1* and their epigenetic modifications. Understanding the epigenetic patterns in Turner syndrome may also provide insight into the sequence of events causing chromosome gene loss, further identifying risk factors for development of the disorder. Advancements in genetic and molecular technology have opened the door for significant progress in of our understanding of Turner syndrome, and we may be on a major innovative threshold.

## Figures and Tables

**Table 1:** Comparison of rates of cardiovascular pathologies in Turner syndrome vs. the general population [4,21].

Cardiovascular Defect	Prevalence in Turner Syndrome	Prevalence in General Population
Bicuspid aortic valve	15–30%	1–2%
Coarctation of the aorta	7–18%	0.04%
Atrial septal defect	1–2%	0.1–0.2%
Ventricular septal defect	1–4%	0.2–0.6%
Partial anomalous pulmonary venous drainage	13–15%	0.4–0.7%
Persistent left superior vena cava	8–13%	0.3–0.5%
Aortic aneurysm	23%	1–2%
Aortic dissection	100-fold increased risk	5–30 cases per million person-years
